# Uterine smooth muscle tumors of uncertain malignant potential: a 13-year retrospective study

**DOI:** 10.3389/fonc.2024.1458968

**Published:** 2024-11-07

**Authors:** Liuliu Liu, Zhendong Xiao, Zhiwen Li, Jinyu Zheng, Xiaofeng Xu, Huaijun Zhou

**Affiliations:** ^1^ Department of Gynecology, Nanjing Drum Tower Hospital, The Affiliated Hospital of Nanjing University Medical School, Nanjing, China; ^2^ Department of Pathology, Nanjing Drum Tower Hospital, The Affiliated Hospital of Nanjing University Medical School, Nanjing, China

**Keywords:** pathological diagnosis, myomectomy, hysterectomy, recurrence, fertility outcome

## Abstract

**Objective:**

The primary objective of this study was to provide valuable evidence for the management of patients diagnosed with uterine smooth muscle tumors of uncertain malignant potential (STUMP), with a focus on those with reproductive aspirations.

**Methods:**

We conducted a retrospective analysis of clinical and pathological data from the medical records and slides of STUMP patients treated at Drum Tower Hospital, affiliated with Nanjing University Medical School, from January 2009 to December 2021.

**Results:**

Thirty-four patients were included in the study, with a median follow-up duration of 76 months (range: 13-157 months). After slide review, the diagnosis agreement rate was 77.3% (34/44 among initially considered cases). The consistency rate between our hospital’s diagnosis and those of other institutions was 75% (15/20). The accuracy rate of intraoperative frozen section diagnosis was low, at 21.4% (3/14). Half of the patients (17) underwent myomectomy, while the other half (17) received hysterectomy, including one subtotal hysterectomy. Two recurrences were observed (5.9%), one as STUMP and the other as leiomyosarcoma, with one recurrence in each surgical group. Notably, 4 of 9 patients with reproductive aspirations successfully underwent cesarean deliveries. Patients with single lesions appeared to exhibit potentially favorable fertility outcomes compared to those with multiple lesions.

**Conclusion:**

The diagnosis of STUMP was difficult. Myomectomy potentially could serve as an alternative for patients with reproductive needs. In selected cases with single lesions, it may indicate potentially favorable fertility outcomes.

## 1 Introduction

Uterine smooth muscle tumors are the most prevalent neoplasms in the female genitourinary system. The prognosis of these tumors is determined by three pivotal factors: cytologic atypia, tumor cell necrosis, and mitotic index. Benign leiomyoma is typically characterized by a low mitotic index and the absence of moderate to severe cytologic atypia or necrosis. Conversely, tumors exhibiting any two or all three of the following features diffuse moderate to severe cytologic atypia, tumor cell necrosis, and a high mitotic index are usually classified as leiomyosarcomas. Uterine smooth muscle tumors of uncertain malignant potential (STUMP) refer to tumors that exceed the morphological criteria for leiomyoma or its variants but do not fulfill the diagnostic criteria for leiomyosarcoma (1). This type of tumor is particularly concerning, particularly for young women who have been diagnosed with STUMP following myomectomy and wish to preserve their reproductive function, as it may progress or potentially represent a missed sarcoma. The incidence of STUMP is relatively low, and the scarcity of large-scale studies has limited the data available for effective management. Therefore, the aim of this research is to investigate the clinical and pathological characteristics of STUMP in our institution, in order to provide valuable insights and evidence for the management of women with reproductive needs.

## 2 Materials and methods

### 2.1 Case selection

We retrospectively analyzed the data of the patients who were diagnosed with STUMP and underwent surgical treatment at the Gynecology Department of Drum Tower Hospital, a tertiary referral center affiliated to Nanjing University Medical School, between January 2009 and December 2021.

### 2.2 Slide review process

All slides were reviewed independently by two senior pathologists specializing in gynecologic oncology. The pathologists were blinded to the patient’s age, original diagnosis, treatments, and recurrence status. The review process was performed using conventional hematoxylin and eosin-stained slides, excluding supplementary immunohistochemical investigations. Tumor cellularity, cytologic atypia, mitotic activity, the presence of any necrosis, epithelioid/myxoid differentiation, atypical mitotic figures, vascular invasion, and infiltrative margins were observed during the slide review. The degree of cellularity and cytologic atypia was categorized as mild, moderate, or severe. The tumor mitotic activity was determined using the highest count method in 10 high-power fields (HPFs) at 400 times magnification. Necrosis was classified as coagulative tumor cell necrosis (CTCN), hyaline necrosis, and uncertain type necrosis.

According to the 2020 World Health Organization (WHO) guideline on STUMP classification (1) and our interpretation of relevant literature (2), the following subcategories were adopted for the diagnosis of STUMP: 1)tumors exhibiting focal/multifocal or diffuse nuclear atypia, with 2-4 mitoses per square millimeter (equivalent to 6-9 mitoses per 10 HPFs), without necrosis; 2)tumors displaying necrosis, such as unequivocal tumor cell necrosis or uncertain type necrosis, but lacking any other concerning features; 3)tumors with mitoses exceeding 6 per square millimeter (equal to ≥15 mitoses per 10 HPFs), but lacking cytologic atypia and tumor cell necrosis; 4)epithelioid or myxoid differentiated tumors falling below the threshold for leiomyosarcoma, yet exceeding the criteria for leiomyoma; 5)tumors infiltrating the myometrium without exhibiting other features of malignancy; and 6) tumors with atypical mitotic figures without any features of malignancy.

### 2.3 Clinical and follow-up data

The patient’s age, parity history, surgical history, symptoms, maximum tumor diameter, surgical procedure, pathological features, recurrence status, and other pertinent information were extracted from medical records. Additionally, all patients underwent pelvic ultrasound examination within six months prior to the study and chest computed tomography (CT) scans within twelve months. Recurrence was defined as the local or distant reappearance of the tumor (either as STUMP or leiomyosarcoma) subsequent to myomectomy or hysterectomy. Importantly, the emergence of benign lesions was not classified as recurrence. Consequently, recurrence must be pathologically confirmed, and new lesions solely detected by imaging studies, without pathological confirmation, were not considered as recurrent disease.

### 2.4 Statistical analysis

S Statistical analysis was conducted using SPSS version 26.0 software. The Kolmogorov-Smirnov test was applied to assess the normality of the data. The nonnormal distribution parameters were expressed as the median and range for continuous variables. Categorical variables were presented as numbers and percentages.

## 3 Results

**Figure 1:**
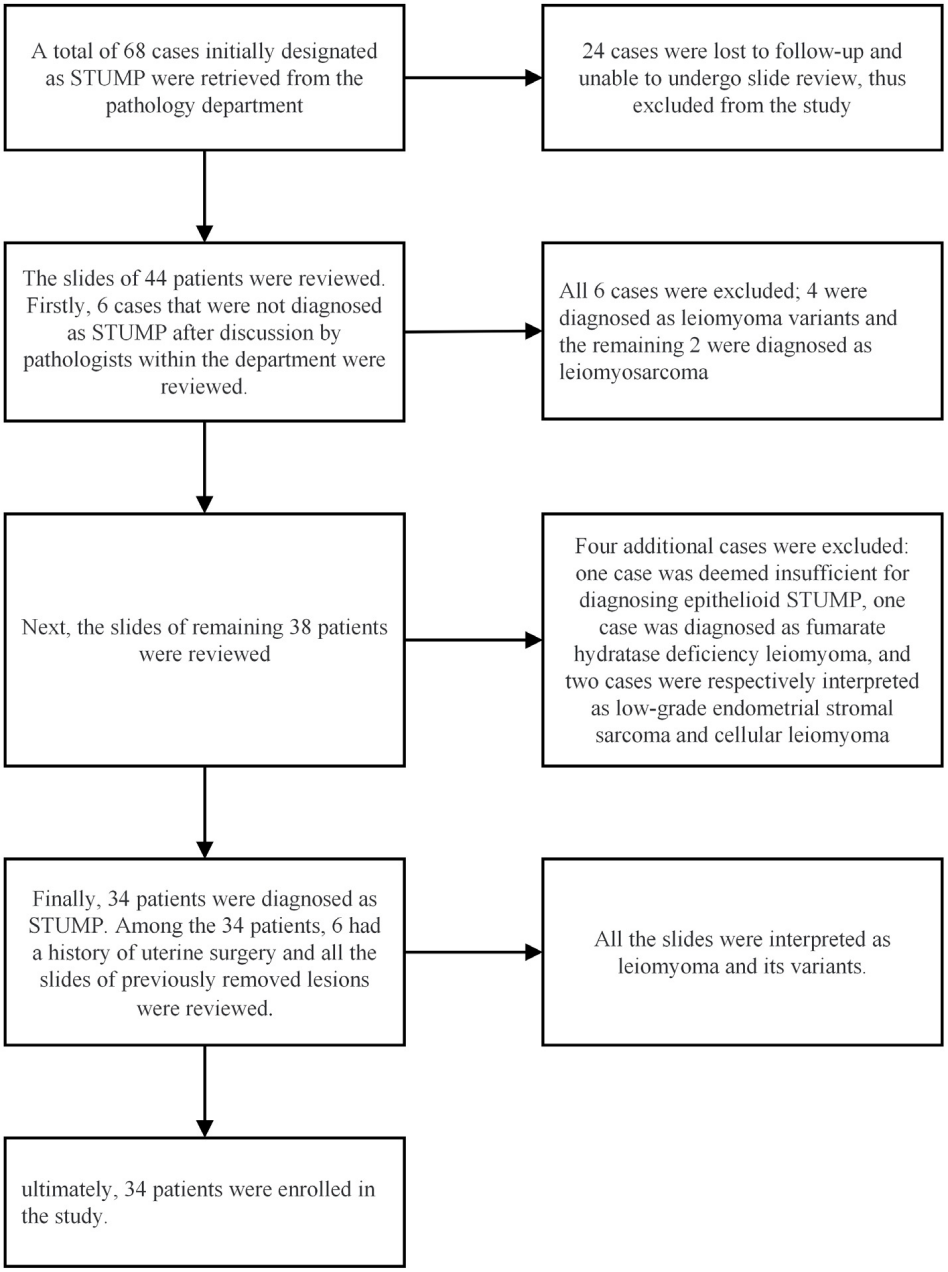
The process of pathological review and case enrollment for the study.

A total of 68 cases were retrieved from the Department of Pathology. Among them, 24 cases were lost to follow-up, leaving 44 cases available for the slide review process. After the slide review, ten patients were excluded due to not meeting the diagnostic criteria for STUMP. Ultimately, 34 patients were enrolled in the study, and their clinicopathological data were meticulously collected. The specific process of the pathological review is illustrated in **Figure 1**.

### 3.1 Clinical characteristics

The patients’ demographic and clinical characteristics are presented in **Table 1**. The median age of the patients was 43.5 years (range: 26–51 years). One patient (2.9%) was in the postmenopausal state, while the remainder were premenopausal. There were 6 nulliparous women (17.6%) and 28 parous women (82.4%). Six patients (17.6%) had a history of previous uterine surgery. None of the patients had a documented history of hormone therapy. Menstrual disorders were the predominant manifestation of STUMP, affecting 16 patients (47.1%). The median maximum tumor diameter was 8 centimeters (range: 3.5-16 cm). Additionally, 23 patients (67.6%) had multiple tumor lesions (including leiomyoma lesions), and 15 patients (44.1%) had multiple STUMP lesions. The median follow-up duration was 76 months (range: 13-157 months). Two patients (5.9%) experienced recurrence as STUMP and leiomyosarcoma, respectively, with no deaths reported. Among the 9 patients with reproductive needs, 4 successfully delivered live babies.

**Table 1:** Demographic and clinical characteristics of patients (*n* = 34).

Clinical characteristics	Values
Age (years)	43.5 (26–51)
20-29	5 (14.7%)
30-39	7 (20.6%)
40-49	19 (55.9%)
≥50	3 (8.8%)
Parity (number)	1 (0-3)
Nullipara	6 (17.6%)
Pluripara	28 (82.4%)
Postmenopausal state	
Yes	1 (2.9%)
No	33 (97.1%)
Hormone therapy history	0 (0%)
Previous uterine surgery	6 (17.6%)
Symptoms	
Menstrual disorders	16 (47.1%)
Health examination	11 (32.4%)
Pain	3 (8.8%)
Abdominal mass	1 (2.9%)
Increased urination frequency	1 (2.9%)
Increased stool frequency	1 (2.9%)
Incidentally detected	1 (2.9%)
Maximum diameter (cm)	8 (3.5–16)
Tumor number^a^	4 (1-14)
Single tumor^a^	11(32.4%)
Multiple tumor^a^	23(67.6%)
Multiple STUMP (before a second surgery)	14 (41.2%)
Multiple STUMP (after a second surgery)	15 (44.1%)
A second surgery (within a short interval)	5(14.7%)
Surgical procedure	
Total Hysterectomy+BS	3 (8.8%)
Total Hysterectomy+BSO+lymphadenectomy	1 (2.9%)
Hysteroscopic Myomectomy	1 (2.9%)
Interval between the two operations (days)	9-47
Surgical procedure (after the second surgeries)	
Myomectomy	17 (50%)
Laparotomic myomectomy	10 (29.4%)
Myomectomy+ partial rectectomy	1 (2.9%)
Myomectomy	9 (26.5%)
Laparoscopic Myomectomy	6 (17.6%)
Hysteroscopic Myomectomy	1 (2.9%)
Hysterectomy	17 (50%)
Subtotal Hysterectomy +BS (Laparoscopic)	1 (2.9%)
Total hysterectomy +/- BS or BSO	16(47.1%)
Laparotomic Hysterectomy	6 (17.6%)
Laparoscopic Hysterectomy	9 (26.5%)
Transvaginal hysterectomy	1 (2.9%)
Morcellation	14 (41.2%)
Laparotomic Myomectomy	1 (7.1%)
Laparoscopic Myomectomy	6 (42.9%)
Hysteroscopic Myomectomy	1 (7.1%)
Subtotal Hysterectomy (Laparoscopic)	1 (7.1%)
Total Hysterectomy (Laparoscopic)	5 (35.7%)
Morcellation methods	14 (41.2%)
Power morcellation	10 (71.4%)
Scalpel morcellation	4 (28.6%)
Oophorectomy	2 (5.9%)
Lymphadenectomy	1 (2.9%)
Chemotherapy	1 (2.9%)
Outcome	
Recurrent	2 (5.9%)
STUMP	1 (50%)
Leiomyosarcoma	1 (50%)
New lesions on imaging (no pathological evidence)	4 (11.8%)
Alive with no evidence of disease	28(82.4%)
Death	0(0%)
Fertility Outcome	4/9(44.4%)
Follow-up duration (median, months)	76 (13-157)

Data are n, median (range), or n (%).

BSO, Bilateral Salpingo-oophorectomy; BS, Bilateral Salpingectomy.

a The term tumor number referred to the number of all tumors, including benign leiomyomas and STUMPs.

### 3.2 Pathological characteristics

**Figure 2:**
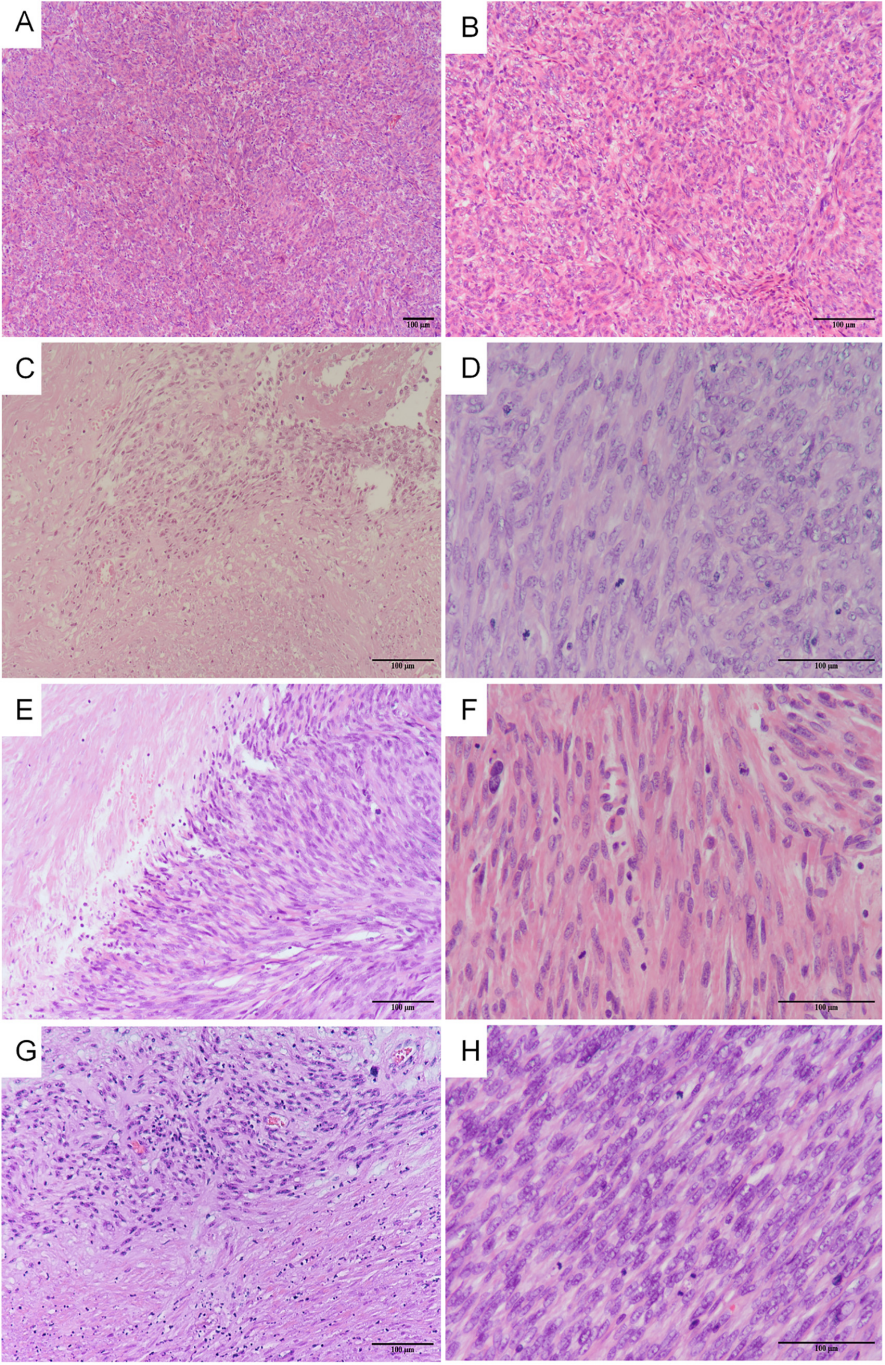
The microscopic findings of the primary and recurrent lesions in the patients with four relapses. The first surgery: **(A)** severe cellularity was found (H&E x 100), **(B)** moderate atypia was found (H&E x 200); The second surgery: **(C)** coagulative tumor cell necrosis was found (H&E x 200), **(D)** mitotic index 10-15 mitoses per 10 HPFs (H&E x 400); The third surgery: **(E)** coagulative tumor cell necrosis was found (H&E x 200), **(F)** mitotic index 10-15 mitoses per 10 HPFs (H&E x 400); The fifth surgery: **(G)** coagulative tumor cell necrosis was found (H&E x 200), **(H)** mitotic index 15-20 mitoses per 10 HPFs (H&E x 400).

In this study, the agreement rate of the initial diagnosis after slide review was 77.3% (34/44). Among the 34 enrolled patients, 25 (73.5%) underwent intradepartmental discussions within the Pathology Department prior to diagnosis. Twenty patients (58.8%) sought pathological consultations in other hospitals, of which 15 (75%) received a diagnosis consistent with ours. Fourteen patients (56%) underwent intraoperative frozen section examination, but only 3 (21.4%) received a diagnosis that concurred with the permanent section diagnosis. **Table 2** summarizes the pathological characteristics of the patients. According to the statistics, 13 cases (38.2%) showed significant cytologic atypia alone, 5 (14.7%) exhibited necrosis alone, 1 (2.9%) demonstrated a high mitotic index alone, 8 (23.5%) displayed at least two Stanford parameters, and 7 (20.6%) did not exhibit any of the three parameters. Consequently, the majority (approximately 79.4%) of STUMP diagnoses were still reliant on these three criteria. The microscopic findings of the primary and recurrent lesions in the patient with four recurrences were illustrated in **Figure 2**. Notably, the microscopic analysis of the lesions obtained during the fourth surgery is not available for presentation, as the surgical procedure was performed at a separate hospital.

**Table 2:** The pathological Characteristics of patients (n = 34).

Pathological Characteristics	Value
Discussion within pathology department	25 (73.5%,25/34)
Consultation in other hospitals	20 (58.8%,20/34)
Consistent diagnosis	15 (75%,15/20)
Intraoperative frozen procedure	14 (41.2%,14/34)
Consistent diagnosis	3 (21.4%,3/14)
Cellularity	
Mild	14 (41.2%)
Moderate	8 (23.5%)
Severe	12 (35.3%)
Atypia	
Mild	13 (38.2%)
moderate	10 (29.4%)
Severe	11 (32.4%)
Mitosis	
0-4	17 (50%)
5–9	13 (38.2%)
10 -14	3 (8.8%)
15-19	0 (0%)
≥20	1 (2.9%)
Necrosis	
No necrosis	23 (67.6%)
Necrosis of uncertain type	9 (26.5%)
Coagulative tumor cell necrosis	2 (5.9%)
Atypical mitotic figures	3 (8.8%)
Tumor infiltrates the myometrium	5 (14.7%)
Epithelioid differentiated	6 (17.6%)
Epithelioid STUMP	0 (0%)
Myxoid differentiated	4 (11.%)
Myxoid STUMP	1 (2.9%)

Data are n, median (range), or n (%).

### 3.3 Treatment and oncologic outcomes

Initially, 13 patients underwent hysterectomy, while 21 patients underwent myomectomy. Shortly after being diagnosed with STUMP, five patients underwent a second operation, and in one patient, residual STUMP lesions were observed. Ultimately, the treatment distribution was even, with 17 patients (50%) undergoing myomectomy and 17 patients (50%) undergoing hysterectomy. Among the 17 patients who underwent hysterectomy, 16 (94.1%) underwent total hysterectomy, while one (5.9%) underwent subtotal hysterectomy. One patient, due to postmenopausal status, underwent additional bilateral salpingo-oophorectomy, and another underwent additional bilateral salpingo-oophorectomy and lymphadenectomy following an intraoperative frozen section diagnosis of sarcoma. One patient, who was considered to have uterine sarcoma during consultation at an external hospital, underwent six courses of chemotherapy there. The remaining patients did not receive chemotherapy, radiotherapy, or endocrine therapy as part of their initial treatment.

**Table 3** summarizes the clinical characteristics of patients in both the myomectomy and hysterectomy groups. Notably, patients in the myomectomy group tend to be relatively younger, with a higher proportion of those who have not yet given birth. Among the 17 patients who underwent myomectomy, 8 underwent morcellation, whereas 6 of the 17 patients in the hysterectomy group underwent similar morcellation procedures. In terms of recurrence, one recurrence case was observed in both the myomectomy and hysterectomy groups, and currently, no statistically significant difference in recurrence rates can be discerned.

**Table 3:** Differences between hysterectomy and myomectomy groups.

	Myomectomy (n=17)	Hysterectomy (n =17)
Age (years)	37 (26-46)	46 (34-51)
Max tumor diamiter(cm)	7.0 (5-10)	8 (3.5-16)
Parity		
Nullipara	6	0
Parous	11	17
Tumor number ^a^		
Single	7	4
Multiple	10	13
Surgical approach		
Open	10	6
No-open	7	11
Morcellation		
Yes	8	6
No	9	11
Recurrence		
Yes	1	1
No	16	16

Data are n, median (range), or n (%).

a The term tumor number referred to the number of all tumors, including benign leiomyomas and STUMPs.

During the study period, 2 patients (5.9%) relapsed with STUMP and leiomyosarcoma, respectively. Their clinical courses are detailed in **Table 4**. Additionally, the clinicopathological characteristics of patients without and with recurrence are presented in **Table 5**. We have not yet observed any statistically significant difference in the surgical approach between recurrent and non-recurrent patients. However, it is noteworthy that both recurrent patients had undergone morcellation. Furthermore, imaging studies detected new uterine lesions in four patients (11.8%), although these lesions were not confirmed pathologically. The remaining 28 patients (82.4%) are alive with no evidence of disease.

**Table 4:** The clinicopathological characteristics of the recurrent patients.

Case	Age	Parity	Time interval since last surgery (month)	Tumor location	Surgery procedure	Adjuvant therapy	Morcellation	Pathological features					Outcome	Follow up (month)
								Diagnosis	Cellularity	Atypia	Mitotic counts	Necrosis		
1	46	2	Initial	Uterus	LSH	No	Yes	STUMP	Severe	Moderate	5-7	uncertain type		
			32	PC	BSO PMR Trachelectomy	No	No	LMS	Severe	Moderate	10-15	CTCN		
			22	PC Omentum	PMR Omentectomy	CT	No	LMS	Severe	Severe	10-15	CTCN		
			18	PC	PMR	CT	No	LMS^a^	–	–	–	–		
			24	PC Sigmoid colon	partial Sigmoidectomy PMR	CT	No	LMS	Severe	Moderate	15-20	CTCN	Alive	110
2	26	0	Initial	Uterus	Myomectomy partial rectectomy	No	Yes	STUMP	Severe	Moderate	1-2	Absent		
			14	Uterus PC AC Omentum	CS, SAH USO PMR Omentectomy	No	No	STUMP	Severe	Severe	1-3	Absent	Alive	56

PC Pelvic cavity, AC Abdominal cavity, LSH Laparoscopic subtotal hysterectomy, BSO Bilateral salpingo-oophorectomy, PMR Pelvic mass resection, CS cesarean section, SAH subtotal abdominal hysterectomy, USO Unilateral salpingo-oophorectomy, CT Chemotherapy, LMS leiomyosarcoma, CTCN coagulative tumor cell necrosis.

a The microscopic findings of lesions from the forth surgery was not presented, because the procedure was performed in another hospital.

**Table 5:** Differences of the clinical and pathological features between no recurrence and recurrence groups.

Characteristics	No recurrence (n = 32)	Recurrence (n =2)
Age (years)	43.5 (27, 51)	36(26, 46)
Parity		
Nullipara	5	1
Pluripara	27	1
Menopausal state		
Yes	1	0
No	31	2
Uterine history		
Yes	6	0
No	26	2
Max tumor diameter	8 (3.5,13)	12.5(9,16)
Tumor number		
Single tumor	10	1
Multiple tumor	22	1
Surgical procedure		
myomectomy	16	1
Hysterectomy	16	1
Morcellation		
Yes	12	2
No	20	0
oophorectomy		
Yes	2	0
No	30	2
Follow-up (months)	76(13,157)	83(56,110)
Cellularity		
Mild	14	0
Moderate	8	0
severe	10	2
Atypia		
Mild	13	0
Moderate	8	2
severe	11	0
Mitosis		
0-4	16	1
5–9	12	1
10–14	3	0
15-19	0	0
≥20	1	0
Necrosis		
No	22	1
Uncertain type	8	1
CTCN	2	0

Data are n, median (range), or n (%).

CTCN, coagulative tumor cell necrosis.

### 3.4 Reproductive outcomes

During the entire follow-up observation period, 4 out of 9 patients with reproductive needs successfully delivered live babies. All 4 of these patients underwent cesarean section deliveries. Among them, one patient experienced an iatrogenic premature delivery at 36 weeks gestation due to complications stemming from the recurrence of STUMP. The remaining three deliveries were full-term cesarean sections. The clinical and pathological characteristics of these 4 patients are presented in **Table 6**. Additionally, among the 9 patients with reproductive needs, 5 had single lesions, while 4 had multiple lesions (specifically, 2, 6, 8, and 10 lesions, respectively). Notably, the live birth rate among patients with single lesions was 80% (4/5), whereas the live birth rate among patients with multiple lesions was 0% (0/4).

**Table 6:** Clinicopathological characteristics of patients with obstetrics outcomes.

Case	Age	Tumor number	Maximum tumor diameter	Parity	Surgical procedure	Pathological features				Timing of delivery	Mode of delivery	Outcome	Supplementary instruction
						Cellularity	Atypia	Mitosis	Necrosis				
1	37	1	10	1	Laparoscopic myomectomy	Moderate	Mild	3-4	Absent	Mature	CS	ANED	A second child was delivered after surgery
2	29	1	5	0	Hysteroscopic myomectomy	Severe	Mild	7	Absent	Mature	CS	ANED	
3	26	1	9	0	Laparotomic myomectomy and partial rectectomy	Severe	Moderate	1-2	Absent	Premature	CS	ANED	Recurrence of disseminated stump during pregnancy
4	29	1	5	0	Laparotomic myomectomy	Moderate	Severe	6	Absent	Mature	CS	ANED	

CS cesarean section, ANED alive with no evidence of disease.

## 4 Discussion

In this 13-year retrospective study, we conducted a thorough analysis of the clinicopathological features of patients diagnosed with STUMP, revealing several meaningful findings. The diagnosis of STUMP poses challenges, characterized by a certain level of variability in pathological diagnoses among different pathologists and institutions, and the intraoperative frozen section accuracy rate remains very low. It appears that myomectomy may not increase the risk of recurrence, and myomectomy potentially could serve as an alternative for patients with fertility aspirations. Patients with a single lesion seem to exhibit potentially favorable fertility outcomes.

The term “STUMP” was first proposed by Kempson et al. to denote a specific group of tumors that exhibit concerning clinical malignant behaviors, yet do not meet the criteria for classification as sarcomas (3). In 1994, researchers at Stanford University conducted a study on 213 cases of suspected uterine smooth muscle tumors, where they initially proposed four histopathological diagnostic criteria for STUMP (4). Subsequently, other researchers proposed their own sets of pathological diagnostic criteria for STUMP (2, 5, 6). Gupta emphasized that histological parameters, including atypical mitosis, epithelioid differentiation, infiltrative or irregular margins, and vascular intrusion, suggest a poor prognosis and should be incorporated into the diagnostic framework for STUMP (2). The latest WHO guidelines have elaborated on the diagnostic criteria for STUMP but emphasized that these criteria were not meant to be taken as doctrine (1). Currently, there exists no uniform histopathological diagnostic criteria for STUMP. Therefore, the diagnosis of STUMP poses a significant challenge, and the reproducibility of diagnoses among different pathologists or institutions remains low. In the largest study conducted to date, which initially included 103 patients, 87 cases met the STUMP diagnostic criteria upon pathological review, yielding a preliminary diagnostic agreement rate of 87.9% (87/99, with 4 cases unavailable for pathological review) (7). In contrast, Basaran et al. reported a lower preliminary diagnostic agreement rate of 71.4% after pathologist review, with 21 patients ultimately diagnosed with STUMP (8). Furthermore, in a European study involving 12 tertiary centers, 29 patients initially diagnosed with STUMP underwent central review by two experienced gynecological pathologists, and only 7 (24.1%) patients ultimately met the diagnostic criteria for STUMP, highlighting an even lower rate of reproducibility (9). In our case series, the majority of cases underwent intra-departmental discussions, resulting in a preliminary diagnostic agreement rate of 77.3% (34/44) after slide review. Among the 20 patients who had prior consultations in other hospitals, 75% (15/20) had consistent diagnostic results with ours, which underscores the difficulties in diagnosing STUMP. The difficulties in pathological identification of these tumors may lead to misdiagnoses, potentially resulting in unnecessary interventions, anxiety, or missed diagnoses of sarcomas. Consequently, we believe that multi-center consultations are worthwhile for STUMP patients due to the significant variations in actual diagnoses.

The diagnostic accuracy and clinical significance of frozen section procedures in the diagnosis of uterine smooth muscle tumors have been evaluated in some studies, but few studies have assessed their application in STUMP (10). Ha et al. reported that among patients with STUMP, 54.5% (6/11) were misdiagnosed as having benign leiomyoma by frozen section procedures, 18.2% (2/11) were misdiagnosed as uterine leiomyosarcoma, and only 27.3% (3/11) were accurately diagnosed as STUMP (11). In our case series, 14 patients underwent intraoperative frozen section procedures. Among them, 9 patients (64.3%) were misdiagnosed as having benign leiomyoma, 2 (14.3%) were misdiagnosed as leiomyosarcoma, and only 3 (21.4%) were correctly diagnosed as STUMP. The accuracy of intraoperative frozen section is very low, and its results should not be used as a basis for hysterectomy, especially for patients who desire to preserve their reproductive function. Furthermore, even in cases with permanent pathological outcomes, seeking pathology consultation from experienced centers should be considered as the first step in the management of the stump.

In addition to multicenter consultation, the expression of immunohistochemical markers, such as estrogen receptor (ER), progesterone receptor (PR), p53, p16, and Ki67, is helpful for pathologists to differentiate leiomyosarcoma, leiomyoma, and STUMPs. Several studies have noted that PR and ER expression are frequently present in STUMP and LM but occurre much less frequently in LMS (12–14). Multiple immunohistochemical studies have observed Ki-67, P53, P16 at high levels in LMS, but it was significantly less frequent or weakly positive in STUMP and LM (15–19). The results of the above studies indicated that the expression of the immunohistochemical markers in STUMP are more comparable to that of LM and LM variants than to that of LMS. The major contribution of immunohistochemical studies may thus be limited in differentiating STUMP from LMS. Inrecent years, molecular studies of uterine smooth muscle tumors have achieved remarkable progress and it may provide a reliable basis for our diagnosis in the future (9, 20–23).

In the current study, the median age of patients diagnosed with STUMP was 43.5 years old, indicating that STUMP tends to occur in relatively younger patients, a finding that aligns with previous literature (7, 24–26). Consequently, uterine-preserving surgery emerges as a potentially favorable option for a substantial proportion of patients, particularly those with reproductive aspirations. Our analysis revealed that among 17 cases of myomectomy and an equal number of hysterectomies, one recurrence was recorded in each group, which appears to indicate that there may be no significant difference in recurrence rates between the two surgical modalities. Similarly, a study conducted by Guntupalli et al. at M.D. Anderson Cancer Center in 2009 observed comparable recurrence rates between their myomectomy group (0/10) and hysterectomy group (3/31) (6). Furthermore, another investigation encompassing 57 patients found that, while 2 recurrences were documented among 30 patients undergoing hysterectomy, 6 recurrences were noted in the 27 patients who underwent myomectomy, however, statistical analysis failed to detect a significant difference in recurrence rates between these two surgical strategies (25). A systematic review involving 189 patients showed that 15 of 62 patients who underwent myomectomy experienced recurrence, and 22 of 127 patients who underwent hysterectomy had recurrence (24). However, no statistically significant difference in recurrence rates was found between the two groups. It appears that myomectomy may not increase the risk of recurrence, and myomectomy potentially could serve as an alternative for patients with fertility aspirations. Currently, after fully evaluating the trade-off between the risk of recurrence and the possibility of fertility, we believe that myomectomy could be a feasible treatment option for young women with reproductive needs.

Numerous studies have been conducted in an effort to identify high-risk factors associated with recurrence. Shim et al. demonstrated that a previous history of myomectomy for leiomyoma was the sole independent risk factor for recurrence (27). Sahin et al. found that the location of the tumor was closely related to recurrence, with the risk of recurrence for subserosal STUMP being 5.72 times higher than that for STUMP located between the muscular walls and the submucosa (25). Travaglino et al. considered significant atypia and CTCN as independent risk factors for recurrence of STUMP (28). Huo et al. showed that mitosis in the initial pathology (mitotic index > 10/10 HPFs) was the only independent risk factor for recurrence (26). Travaglino et al. found that an abnormal p53 expression and a diffuse p16 expression were significantly associated with the risk of recurrence of STUMP (29). To date, no consensus has been reached regarding the specific risk factors associated with relapse. However, a systematic review published in 2022 showed that unprotected morcellation of lesions was significantly associated with disease recurrence (24). Actually, as early as 2014, the United States Food and Drug Administration issued an alert that focused the entire world’s attention on the relevant risks. My study did not identify any significant high-risk factors for recurrence, but the fact that both recurrent cases underwent morcellation suggests that morcellation may be a potential risk factor for recurrence. Therefore, based on existing literature, we recommend avoiding unprotected morcellation due to its association with increased risks of recurrence and metastasis (7, 24, 30–32). Given the challenges in preoperative and intraoperative diagnosis of STUMP, we advise against unprotected morcellation for all patients with uterine smooth muscle tumors.

Among STUMP patients who underwent fertility-sparing surgery, the reported successful pregnancy rate ranged from 20% to 80%, with favorable maternal and infant outcomes (33). Our study revealed that patients with STUMP had a decent chance of fertility (44.4%, 4/9). Sahin reported that ten of 27 patients who underwent myomectomy for uterine myoma had fertility desire, with seven(70%,7/10) recorded pregnancies (25). While Huo reported a successful pregnancy rate of 20% (7/35) among patients attempting to conceive (26). In our study, the live birth rate among patients with single lesions was 80% (4/5), whereas the live birth rate among patients with multiple lesions was 0% (0/4). It seems that patients with a single lesion exhibit potentially favorable fertility outcomes and may therefore be more suitable candidates for uterine preservation surgery. We suggest that caution should be exercised when choosing fertility-sparing surgery for patients with multiple lesions.

## 5 Strengths and limitations

The most definitive advantage of our study is that the pathology of all lesions has been reviewed, thereby ensuring the homogeneity of case quality. Secondly, the follow-up period is relatively long. We particularly paid attention to the concordance rate of pathological results among different centers, as well as the concordance rate between intraoperative frozen sections and permanent sections. The main limitations of this study lie in its retrospective nature, relatively small sample size, and the wide span of follow-up time.

## 6 Conclusion

In conclusion, the diagnosis of STUMP was difficult, and multicenter consultation was recommended to distinguish malignant sarcoma. Myomectomy appears an alternative for patients with fertility aspirations. Patients with a single lesion seem to exhibit potentially favorable fertility outcomes.

## Data Availability

The data analyzed in this study is subject to the following licenses/restrictions: The datasets for this article are not publicly available due to patient privacy concerns. Requests to access the datasets should be directed to liu679626@163.com.

## Glossary

STUMP: smooth muscle tumors of uncertain malignant potential
HPFs: High-power fields
CTCN: Coagulative tumor cell necrosis
WHO: World Health Organization
CT: Computed tomography
LMS: Leiomyosarcomas
LM: Leiomyoma
PR: Progesterone receptor
ER: estrogen receptor
H&E: Hematoxylin and eosin-stained
BSO: Bilateral Salpingo-oophorectomy
BS: Bilateral Salpingectomy
PC: Pelvic cavity
AC: Abdominal cavity
LSH: Laparoscopic subtotal hysterectomy
PMR: Pelvic mass resection
CS: Cesarean section
SAH: Subtotal abdominal hysterectomy
USO: Unilateral salpingo-oophorectomy
ANED: alive with no evidence of disease.
